# Response to: Comment on “Original Solution for Middle Ear Implant and Anesthetic/Surgical Management in a Child with Severe Craniofacial Dysmorphism”

**DOI:** 10.1155/2016/8354034

**Published:** 2016-12-05

**Authors:** Giovanni Bianchin, Lorenzo Tribi, Aronne Reverzani, Patrizia Formigoni, Valeria Polizzi

**Affiliations:** ^1^MD Otolaryngology and Audiology Department, Santa Maria Nuova Hospital, Viale Risorgimento, No. 80, 42100 Reggio Emilia, Italy; ^2^MD Emergency Medicine Department, Santa Maria Nuova Hospital, Viale Risorgimento, No. 80, 42100 Reggio Emilia, Italy

We thank Kruyt et al. [1], for their interest in our publication titled “Original Solution for Middle Ear Implant and Anesthetic/Surgical Management in a Child with Severe Craniofacial Dysmorphism” [2]. In response to their comments, we would like to offer clarification as to the choice of treatment for our five-year-old patient with Van Maldergem syndrome, affected by severe bilateral malformation of the external auditory canal and middle ear.

It is important to reiterate the fact that we are independent authors working for a public hospital. We do not have any conflict of interests nor are we financially supported by any company.

The goal of our case report was to present an example of a multidisciplinary approach for the treatment of congenital aural atresia with severe craniofacial dysmorphism. As stated in the article, the multidisciplinary team was formed by speech therapists, audiologists, medical doctors, neuropsychiatrists, anaesthesiologists, surgeons, radiologists, and, of course, the parents. In our article, we aimed to emphasize the importance of a multidisciplinary approach, as there are currently no guidelines available for syndromic children. Only after a careful evaluation of the pros and cons of all available treatment options, including those the patient had already trialled, was a consensus reached. The parents themselves rejected percutaneous bone conduction devices for their child, not because of the (suboptimal) pre-op hearing impression or discomfort of the test steel spring headband but due to the permanent wound and the aesthetics of the externally worn device. The bone conductor trial showed more than satisfactory results.

As the child's malformation has a grade of 6 on the Jahrsdoerfer score, surgical reconstruction of the EAC was not considered a possibility. Publications report that patients with a higher score than 6 have a significantly better hearing outcome after surgery [3].

Bone-anchored hearing aids have been considered as first option [4]. A literature review looking into postoperative complications with the percutaneous bone conduction implants confirms a high incidence of complications related to the percutaneous system.

What follows are outcomes from a systematic study review by Kiringonda and Lusting [5], illustrating the complication rates from bone conduction hearing aid.

The article analysed 20 articles and 2,134 patients who underwent a total of 2,310 osseoimplants. Failure of osseointegration ranged from 0% to 18% in adult and mixed populations and 0% to 14.3% in the paediatric population. The rate of revision surgery ranged from 0.0% to 44.4% in pediatric patients, whereas the total rate of implant loss ranged from 0.0% to 25% in pediatric patients.

Ernst et al. [6] reported postoperative complications with percutaneous bone conductions devices as well: in a total of 543 patients who received 609 implants, the occurrence of adverse skin reactions of grade 1 or 2 according to Holger's grading was as high as 29.4%. Revision surgery was required in 29.9%.

In addition to the above outcomes, scientific evidence proves increased complication rates following paediatric percutaneous bone conduction device surgery [7–11]. Loss of the fixture due to failure of osseointegration is significantly higher in younger children [9, 12]. Compromised bone quality or immature and abnormal bone structure presents an additional burden to osseointegration of the screw. Cass and Mudd (2010) [13] consider this a relative contraindication for a percutaneous bone conductor implant. The presented case is made more complex by the fact that our patient is not only a child but is also suffering from a syndrome. Recent literature shows that complication rates are particularly common in syndromic children [14–19]. The skull bone thickness was less than 3 mm (measured on the axial CT slices at 1 cm posterior to the sigmoid sinus, at the superior margin of the bony canal). As trauma to the head is always a possibility and the bone is very thin, the additional risk of percutaneous bone conduction implants causing intracranial intrusion of fixture or other severe risks also needed to be considered [20–23].

Due to the increased incidence of complications with percutaneous bone conduction devices mentioned above and due to the parent's decision, our multidisciplinary team opted to look into intact skin solutions such as the Vibrant Soundbridge.

An important aspect considered has been binaural hearing. The cranial malformation of the 5-year-old affects hearing on both sides. The transducer of the VSB stimulates the only implanted ear. Therefore a contralateral implantation could reestablish a bilateral hearing sensation.

Another key aspect is the wide amplification range of middle ear transducers. It is shown that the Vibrant Soundbridge provides high gain especially in the high frequencies leading to better speech comprehension in noise [24, 25].

We would also like to comment on the reversibility of the performed treatment and the risk of the anesthesia.

The Vibrant Soundbridge is a reversible procedure as no structures of the middle ear have been harmed. The intervention is not compromising any future treatment opportunity including the aesthetical reconstruction of the auricle. We mentioned in the article that the surgery was performed respecting the skin needed for auricle reconstruction. Special care was taken while performing the skin incision in order to enable the aesthetic surgeons to reconstruct the auricle [26].

The additional risk of anaesthesia for maxillofacial malformations is related to the intubation itself, while no additional risk is represented by the prolonged duration of the surgery. In our hospital, all implantable devices require intubation. Therefore the chosen treatment did not increase the risk for the patient.

Regarding MRI incompatibility, in the last years, results from extensive testing for MRI safety were published and a 1.5 T MRI examination can be performed on VSB users at a calculated risk [27]. The patient's parents were extensively informed of the possibility of transducer dislocation after exposure to a magnetic field over 1.5 T, and the choice to proceed with an MRI was made with all of the risks considered. If the patient will undergo an MRI examination image artefacts will be seen in proximity of the implant. This is a known issue that even cochlear implant users need to face. We would like to emphasize that the patient does not have a neurological development disorder or a pathology which would require regular MRIs.
